# Supplementary material for: Promising Antifungal Molecules against Mucormycosis Agents Identified from Pandemic Response Box®: In Vitro and In Silico Analyses
Source: J Fungi (Basel). 2023 Jan 31;9(2):187. doi: 10.3390/jof9020187 (PMC9959553; doi:10.3390/jof9020187)
Supplement: Supplementary file 1 [file jof-09-00187-s001.zip › jof-2149566-supplementary.pdf]

Table S1. Pandemic Response Box® screening data. A total of 400 compounds were tested against *R. oryzae* UCP1295.

Compounds code	% of growth inhibition				Mean	SD
Control - Posaconazole	54.1	60.4	60.4	50.1	56.3	4.4
MMV1634492	-52.4	-15.3	-28.2	-26.3	-30.6	13.5
MMV1782108	-10.5	-16.6	-10.0	-17.6	-13.7	3.4
MMV1634494	-17.2	-13.7	-12.9	-15.5	-14.8	1.6
MMV1634386	-13.9	-11.3	-16.3	-19.4	-15.2	3.0
MMV637528	-31.5	-20.2	-37.1	-18.5	-26.8	7.7
MMV1782109	-4.8	-13.9	-13.7	-23.5	-14.0	6.6
MMV1645051	-44.0	-33.9	-45.6	-34.7	-39.6	5.3
MMV1634491	-18.3	-12.8	-15.5	-13.7	-15.1	2.1
MMV002731	-13.5	-12.4	-13.5	-12.6	-13.0	0.5
MMV000059	-37.1	-37.1	-41.9	-38.1	-38.5	2.0
MMV637104	-32.3	-31.5	-29.0	-29.0	-30.4	1.4
MMV637533	-5.5	-4.3	-8.4	-2.4	-5.2	2.2
MMV1634362	-30.6	-32.7	-48.2	-33.1	-36.2	7.0
MMV1634360	-16.7	-19.0	-18.0	-18.6	-18.1	0.9
MMV002565	-41.1	-37.1	-37.1	-42.7	-39.5	2.5
MMV396785	72.2	70.2	68.5	69.5	70.1	1.3
MMV003143	-14.7	-13.5	-13.9	-14.4	-14.1	0.5
MMV002337	-21.0	-45.2	-41.9	-37.9	-36.5	9.3
MMV1634363	-16.5	-16.7	-18.1	-20.4	-17.9	1.6
MMV1634359	-40.3	-44.4	-50.0	-35.2	-42.5	5.4
MMV1782110	-5.0	-2.0	-2.2	-2.2	-2.8	1.2
MMV1634493	-21.0	-37.9	-27.4	-36.3	-30.7	6.9
MMV344625	-16.7	-12.6	-12.2	-11.5	-13.3	2.0
MMV1634358	-10.4	-16.9	-61.5	-15.0	-26.0	20.6
MMV1578560	-15.6	-12.8	-14.9	-14.9	-14.6	1.0
MMV640014	-24.2	-17.9	-16.9	-12.1	-17.8	4.3
MMV004508	-48.4	-36.3	-22.6	-22.6	-32.5	10.8
MMV1782212	-2.6	-6.6	-7.9	-2.6	-4.9	2.4
MMV1782227	-21.8	-29.0	-16.9	-28.2	-24.0	5.0
MMV1782226	-19.2	-18.9	-27.4	-14.5	-20.0	4.7
MMV1782221	-18.2	-13.6	-13.0	-13.1	-14.5	2.2
MMV1782224	-13.3	-12.4	-13.5	-18.7	-14.5	2.5
MMV1782223	-25.8	-36.0	-16.9	-26.6	-26.3	6.7
MMV1782218	-14.5	-16.1	-26.6	-26.6	-21.0	5.7
MMV1782217	-35.4	-21.5	-32.2	-27.4	-29.1	5.2
MMV1782216	-25.0	-10.2	-18.1	-21.8	-18.8	5.5
MMV1009286	-13.3	-13.5	-13.3	-11.5	-12.9	0.8
MMV1782225	-17.7	-37.9	-21.0	-33.9	-27.6	8.5
MMV1782354	-25.3	-50.1	-51.5	-25.8	-38.2	12.6
MMV689401	-40.3	-21.0	-33.1	-51.6	-36.5	11.1
MMV1782351	-14.2	-15.6	-29.8	-12.1	-17.9	7.0
MMV1782387	-52.4	-27.4	-24.2	-18.5	-30.6	13.0

MMV1782389	-18.1	-16.9	-18.7	-29.0	-20.7	4.9
MMV1782388	-27.7	-34.0	-26.5	-21.9	-27.5	4.3
MMV1634557	-15.2	-14.1	-14.7	-19.4	-15.9	2.1
MMV030787	-5.5	-6.1	-8.2	-0.2	-5.0	3.0
MMV000028	-4.5	-4.3	-5.8	-3.3	-4.5	0.9
MMV002722	-13.7	-19.4	-16.5	-12.8	-15.6	2.6
MMV1593535	-5.2	-1.1	-0.3	-5.2	-3.0	2.3
MMV1593533	-13.1	-12.0	-14.9	-15.0	-13.8	1.3
MMV422940	-11.3	-11.9	-11.1	-12.4	-11.7	0.5
MMV1582497	-18.4	-17.4	-18.2	-14.4	-17.1	1.6
MMV1581553	-24.2	-11.8	-17.7	-29.0	-20.7	6.5
MMV1581548	-12.7	-18.4	-19.2	-13.1	-15.8	3.0
MMV1580850	-22.6	-22.6	-20.2	-38.7	-26.0	7.4
MMV002740	-35.5	-27.4	-21.0	-24.2	-27.0	5.4
MMV1579783	-31.5	-33.9	-41.1	-31.5	-34.5	4.0
MMV1578925	-27.4	-23.4	-31.5	-19.4	-25.4	4.5
MMV1578889	-16.5	-15.1	-14.7	-13.9	-15.1	0.9
MMV1578572	-13.7	-15.6	-13.3	-15.8	-14.6	1.1
MMV1578568	-15.0	-13.9	-15.4	-14.9	-14.8	0.6
MMV1580173	37.9	54.8	47.6	46.8	46.8	6.0
MMV1634403	-18.4	-37.1	-47.6	-47.6	-37.7	11.9
MMV1634390	-16.7	-17.7	-14.6	-13.6	-15.7	1.6
MMV1633967	-29.8	-28.2	-19.4	-37.1	-28.6	6.3
MMV1593534	*-30.0*	-19.8	-14.0	-16.0	-20.0	6.2
MMV1582498	-18.9	-18.4	-15.8	-14.7	-17.0	1.7
MMV1582494	-18.1	-12.8	-14.7	-12.8	-14.6	2.1
MMV1582493	-34.7	-31.5	-29.8	-52.4	-37.1	9.0
MMV000043	-41.9	-36.3	-33.9	-34.7	-36.7	3.1
MMV003137	-11.4	-11.1	-12.2	-12.4	-11.8	0.5
MMV1581552	-10.0	-10.8	-11.9	-15.7	-12.1	2.2
MMV1581551	-10.1	-12.4	-11.4	-11.7	-11.4	0.9
MMV1580853	-50.8	-22.6	-42.1	-20.6	-34.0	12.8
MMV1580841	-49.2	-42.0	-32.6	-46.0	-42.4	6.2
MMV1579878	-40.3	-37.1	-36.8	-53.2	-41.9	6.7
MMV1579845	-18.1	-14.5	-16.5	-13.7	-15.7	1.7
MMV1579787	-34.7	-45.2	-14.7	-25.8	-30.1	11.2
MMV1579786	-18.1	-16.7	-17.8	-14.3	-16.7	1.5
MMV1579784	-35.5	-31.5	-19.2	-23.6	-27.4	6.4
MMV1483032	-15.5	-13.1	-12.2	-11.5	-13.1	1.5
MMV508427	-12.8	-6.9	-2.3	-17.7	-9.9	5.8
MMV1578565	-14.7	-12.3	-16.2	-14.6	-14.4	1.4
MMV1578561	-29.2	-33.8	-12.3	-22.3	-24.4	8.1
MMV1578557	-5.4	-8.5	-5.4	-3.1	-5.6	1.9
MMV000725	-22.3	-23.1	-36.9	-27.7	-27.5	5.8
MMV1634384	-4.6	-4.6	-7.7	-3.1	-5.0	1.7
MMV1634383	-4.0	-3.8	-2.2	-5.4	-3.9	1.1
MMV1613560	-11.5	-18.5	-7.7	-12.5	-12.6	3.9
MMV1230557	-3.8	-4.6	-5.4	-0.8	-3.7	1.8

MMV1593540	-0.3	-0.8	-0.4	-0.9	-0.6	0.3
MMV1593539	-5.4	-0.5	-1.5	-0.8	-2.0	2.0
MMV1582495	-5.5	-10.8	-3.1	-5.4	-6.2	2.8
MMV1582487	-10.4	-11.6	-11.2	-12.8	-11.5	0.9
MMV021759	-4.6	-3.1	0.0	-5.4	-3.3	2.1
MMV1581555	-8.5	-6.9	-5.4	-6.8	-6.9	1.1
MMV1581547	-4.6	-1.5	-2.3	-3.8	-3.1	1.2
MMV687800	-3.1	0.0	-1.5	-2.2	-1.7	1.1
MMV1580848	-16.9	-11.8	-15.4	-18.5	-15.6	2.5
MMV1580842	0.4	0.2	0.7	0.4	0.4	0.2
MMV1580840	-7.7	-8.5	-8.5	-5.4	-7.5	1.3
MMV046261	-8.5	-6.9	-8.5	-11.1	-8.7	1.5
MMV1579846	-10.0	-11.0	-13.0	-12.2	-11.6	1.1
MMV1578842	-0.2	-0.4	0.0	-0.9	-0.4	0.3
MMV1579776	-34.6	-24.6	-22.3	-15.4	-24.2	6.9
MMV1579775	-7.7	-4.6	-7.7	-8.5	-7.1	1.5
MMV1579354	-33.1	-34.6	-26.9	-30.8	-31.3	2.9
MMV1578899	-4.6	-2.5	-2.2	-1.2	-2.6	1.2
MMV1578890	0.2	-0.5	1.2	1.5	0.6	0.8
MMV1578886	-11.0	-10.5	-10.7	-13.1	-11.3	1.0
MMV1578573	-3.8	-9.2	-1.5	0.0	-3.7	3.5
MMV1578571	-8.5	-6.2	-7.0	-7.2	-7.2	0.8
MMV687273	-8.5	-1.5	0.0	-3.1	-3.3	3.2
MMV1634387	-15.4	-18.5	-11.0	-16.9	-15.4	2.8
MMV1633966	-44.6	-23.1	-28.5	-24.6	-30.2	8.6
MMV1505642	-0.2	-4.6	-4.6	-6.9	-4.1	2.4
MMV1593542	-11.5	-15.4	-16.2	-13.1	-14.0	1.9
MMV1593538	-0.5	0.3	0.0	1.6	0.4	0.8
MMV002560	0.0	-10.2	-13.8	-1.6	-6.4	5.8
MMV002459	-17.7	-13.1	-12.9	-12.6	-14.1	2.1
MMV637945	-23.1	-32.3	-31.5	-23.1	-27.5	4.4
MMV1580854	-25.1	-37.7	-41.5	-42.3	-36.7	6.9
MMV1580851	-21.5	-8.3	-16.5	-8.5	-13.7	5.6
MMV1579849	-41.5	-30.8	-16.9	-13.8	-25.8	11.1
MMV1579844	-3.8	-5.4	-1.2	-2.6	-3.3	1.5
MMV141011	-32.3	-10.5	-12.3	-11.5	-16.6	9.1
MMV002516	-18.5	-13.5	-14.6	-17.7	-16.1	2.1
MMV688755	0.9	2.8	0.3	1.0	1.3	0.9
MMV020752	-5.4	-9.0	-10.3	-10.8	-8.9	2.1
MMV1229204	-6.2	-14.6	-13.1	-11.1	-11.2	3.2
MMV1578575	-15.4	-31.5	-11.7	-26.9	-21.4	8.1
MMV1578556	-6.9	-6.2	-3.8	-2.4	-4.8	1.8
MMV1663457	0.0	-5.6	2.6	1.3	-0.4	3.1
MMV1341773	-25.4	-30.8	-36.2	-20.8	-28.3	5.8
MMV1633970	-14.6	-10.8	-10.8	-12.8	-12.3	1.6
MMV1633965	-7.7	-11.8	-13.8	-13.2	-11.6	2.4
MMV093250	-12.3	-16.9	-18.5	-16.2	-16.0	2.3
MMV1593541	-16.9	-13.1	-31.5	-25.4	-21.7	7.2

MMV1593537	-19.2	-6.2	-11.5	-10.0	-11.7	4.8
MMV1582492	-4.6	-6.9	-8.5	-6.9	-6.7	1.4
MMV1582491	-42.3	-21.5	-31.5	-11.5	-26.7	11.4
MMV1582382	-3.8	-16.9	-13.6	-13.5	-12.0	4.9
MMV002354	-21.5	-26.9	-23.1	-18.5	-22.5	3.0
MMV1581557	0.8	5.4	-1.9	-3.1	0.3	3.3
MMV1581554	-43.5	-25.4	-28.5	-20.8	-29.5	8.5
MMV1581550	-41.5	-21.5	-39.2	-16.2	-29.6	11.0
MMV1581546	-7.7	-6.2	-8.5	-1.5	-6.0	2.7
MMV1580844	62.3	80.8	69.2	68.5	70.2	6.7
MMV1579788	-33.8	-24.6	-30.0	-26.9	-28.8	3.5
MMV1579785	-18.5	-14.6	-20.0	-20.0	-18.3	2.2
MMV811071	-17.7	-38.5	-16.9	-13.8	-21.7	9.8
MMV1578898	0.0	1.5	0.0	-0.5	0.3	0.8
MMV1549626	-23.1	-40.8	-21.5	-18.5	-26.0	8.7
MMV1578559	-19.2	-17.7	-27.7	-25.4	-22.5	4.2
MMV1578579	0.0	-4.6	-2.3	-6.2	-3.3	2.3
MMV1578578	-21.5	-36.9	-30.0	-43.1	-32.9	8.0
MMV1578574	-45.4	-48.5	-36.9	-46.2	-44.3	4.4
MMV1578569	-13.8	-15.4	-16.9	-11.9	-14.5	1.9
MMV1578566	-12.3	-26.2	-21.5	-22.3	-20.6	5.1
MMV1578563	-0.5	-0.6	-0.4	-0.5	-0.5	0.1
MMV1578558	-46.9	-34.6	-48.8	-28.9	-39.8	8.3
MMV1782140	-3.7	-5.6	-1.1	-5.9	-4.1	1.9
MMV1634391	-20.5	-18.8	-15.1	-15.3	-17.4	2.3
MMV1633674	-23.4	-19.1	-19.5	-17.4	-19.9	2.2
MMV002169	-9.2	-6.2	-3.8	-2.6	-5.4	2.5
MMV002612	-21.5	-27.9	-15.5	-22.3	-21.8	4.4
MMV1613562	-0.1	-0.6	-0.3	-0.6	-0.4	0.2
MMV689758	-23.2	-29.1	-11.3	-16.2	-19.9	6.8
MMV1593532	-14.7	-14.1	-14.5	-15.7	-14.7	0.6
MMV002224	-3.1	-7.9	-9.8	-4.1	-6.2	2.8
MMV637659	-22.6	-18.6	-19.3	-16.1	-19.1	2.3
MMV1580846	-27.6	-27.6	-37.4	-41.5	-33.5	6.1
MMV1580845	-26.0	-26.0	-57.7	-40.7	-37.6	13.1
MMV1579850	-5.7	-8.1	-1.4	-4.5	-4.9	2.4
MMV374187	-19.9	-18.5	-22.0	-14.7	-18.8	2.6
MMV1579781	-37.4	-26.9	-36.2	-28.7	-32.3	4.6
MMV1579780	-16.4	-12.0	-16.8	-15.5	-15.2	1.9
MMV688756	0.5	-11.1	-8.5	-13.9	-8.2	5.4
MMV1578897	0.9	5.8	1.3	-12.9	-1.2	7.0
MMV001014	-9.1	-3.3	-11.2	-18.7	-10.6	5.5
MMV1578885	-12.4	-16.6	-15.9	-12.2	-14.2	2.0
MMV687696	-30.2	-43.9	-20.4	-39.0	-33.4	8.9
MMV011565	-51.8	-48.0	-42.3	-26.3	-42.1	9.7
MMV452821	-17.0	-16.9	-14.0	-16.3	-16.1	1.2
MMV1578576	-16.7	-43.9	-47.7	-50.4	-39.7	13.5
MMV1578562	-17.2	-14.7	-16.3	-29.3	-19.4	5.8

MMV1578555	-5.0	-39.8	-15.6	-14.1	-18.6	12.9
MMV1578554	-15.4	-14.8	-15.5	-12.3	-14.5	1.3
MMV1634402	-1.5	-9.3	-3.4	-5.2	-4.8	2.9
MMV1634399	-15.0	-16.9	-17.8	-15.5	-16.3	1.1
MMV000051	-39.8	-17.1	-18.5	-43.1	-29.6	11.9
MMV002287	-58.5	-21.4	-32.5	-24.0	-34.1	14.7
MMV831201	-3.3	-6.6	-4.7	-3.7	-4.6	1.3
MMV687801	-12.3	-12.5	-14.3	-13.7	-13.2	0.8
MMV1593531	-56.1	-23.6	-24.1	-13.0	-29.2	16.2
MMV1581558	51.2	41.5	60.2	60.2	53.3	7.7
MMV1581556	-47.2	-28.9	-41.5	-53.7	-42.8	9.1
MMV1581549	20.3	21.1	17.1	16.3	18.7	2.1
MMV1580847	-17.7	-17.5	-16.2	-15.8	-16.8	0.8
MMV1579847	-7.2	-9.6	-5.3	-6.7	-7.2	1.5
MMV1579782	-0.4	-0.8	-5.0	-1.9	-2.1	1.8
MMV1579777	-4.8	-6.5	-6.9	-7.2	-6.3	0.9
MMV292173	-12.4	-16.2	-18.0	-15.3	-15.4	2.0
MMV102270	-55.6	-13.2	-41.4	-22.8	-33.2	16.4
MMV000008	-0.2	-1.8	-12.8	0.8	-3.5	5.4
MMV002260	-20.7	-14.7	-8.9	-17.9	-15.5	4.4
MMV102833	3.6	17.4	-0.7	-13.3	1.7	11.0
MMV565773	-30.9	-19.5	-43.9	-26.0	-30.1	8.9
MMV1578577	-1.8	-1.6	-11.2	-4.8	-4.8	3.9
MMV233495	1.4	1.0	-4.3	0.5	-0.4	2.3
MMV002676	-20.1	-13.4	-17.6	-14.2	-16.3	2.7
MMV1633675	-24.4	-30.9	-27.3	-19.3	-25.5	4.2
MMV303733	-16.3	-3.0	-1.7	-13.5	-8.6	6.4
MMV003249	-5.2	-5.0	-4.7	-5.7	-5.2	0.4
MMV001438	-9.6	-14.6	-14.7	-13.8	-13.2	2.1
MMV1582496	-0.4	0.0	-1.9	-2.8	-1.3	1.1
MMV1582490	-2.3	-6.9	-3.9	1.1	-3.0	2.9
MMV1582489	-14.7	-22.3	-23.3	-14.7	-18.8	4.1
MMV1582488	-5.5	-6.8	-9.0	-8.8	-7.5	1.4
MMV002665	2.0	-9.6	-1.6	0.3	-2.2	4.4
MMV1581559	-19.9	-17.7	-19.1	-18.7	-18.9	0.8
MMV1581545	-55.6	-42.3	-31.5	-37.9	-41.8	8.8
MMV1580855	0.2	-2.0	-1.2	-0.7	-1.0	0.8
MMV1580852	-13.4	-10.4	-15.1	-12.6	-12.9	1.7
MMV1580849	-15.3	-21.5	-20.9	-21.8	-19.9	2.6
MMV1580839	-9.3	-2.3	-0.2	-0.5	-3.1	3.7
MMV1579779	-35.8	-31.8	-35.7	-29.9	-33.3	2.5
MMV1579778	-14.8	-11.1	-12.0	-16.1	-13.5	2.0
MMV099714	-3.0	-7.3	-1.0	0.5	-2.7	2.9
MMV1578891	-12.7	-13.8	-21.9	-15.7	-16.0	3.6
MMV124656	-56.5	-40.3	-22.0	-32.5	-37.8	12.6
MMV975972	1.7	-0.8	0.9	-0.7	0.3	1.1
MMV1578884	-1.0	-1.4	-1.8	-1.2	-1.3	0.3
MMV108465	-20.3	-41.3	-35.8	-31.9	-32.3	7.7

MMV1480967	-19.9	-20.5	-21.5	-19.6	-20.4	0.7
MMV1578570	-17.2	-22.8	-25.8	-25.0	-22.7	3.4
MMV1578564	-17.6	-16.2	-19.0	-16.7	-17.4	1.1
MMV1782215	-5.8	-8.5	-5.0	-5.2	-6.1	1.4
MMV1782403	-44.7	-43.1	-41.5	-41.5	-42.7	1.3
MMV1782402	-25.9	-10.6	-23.5	-19.1	-19.8	5.9
MMV1782411	-1.6	3.3	-15.4	-17.1	-7.7	8.7
MMV214956	-17.9	1.6	-22.0	-33.3	-17.9	12.6
MMV1634395	-10.6	-6.5	-30.1	-35.8	-20.7	12.4
MMV1634071	-7.3	-2.4	-18.7	-31.7	-15.0	11.3
MMV1633962	-11.4	2.4	-23.6	-16.3	-12.2	9.5
MMV1633678	-0.8	1.6	-25.2	-16.3	-10.2	11.1
MMV690480	4.9	8.1	-8.9	-11.4	-1.8	8.5
MMV1593517	-26.0	2.4	-4.1	-6.5	-8.5	10.6
MMV1593515	21.1	34.1	20.3	30.9	26.6	6.0
MMV1581032	-4.9	-8.1	-4.9	3.3	-3.7	4.2
MMV1580799	3.3	-8.1	-2.4	0.8	-1.6	4.3
MMV001726	-16.3	-13.0	-10.6	-23.6	-15.9	4.9
MMV690653	-13.0	-12.2	-3.3	1.6	-6.7	6.1
MMV1580484	-26.8	-10.6	-2.4	8.9	-7.7	13.0
MMV1580478	-35.0	-19.5	-15.4	-8.1	-19.5	9.8
MMV1782102	-13.0	-26.0	-4.1	-1.6	-11.2	9.6
MMV394033	-13.0	-4.9	4.1	10.6	-0.8	8.9
MMV1634404	-0.8	0.0	6.5	10.6	4.1	4.7
MMV1634388	-13.8	-10.6	-4.1	9.8	-4.7	9.0
MMV180402	4.9	4.1	-1.6	4.9	3.0	2.7
MMV642550	62.6	68.3	55.3	52.8	59.8	6.1
MMV1580798	6.5	7.3	-14.6	-9.8	-2.6	9.7
MMV1580800	-1.6	1.6	-13.8	-18.7	-8.1	8.4
MMV1580503	-3.3	5.7	-3.3	-3.3	-1.0	3.9
MMV690547	-4.9	9.8	-12.2	-7.3	-3.7	8.2
MMV1165877	-2.4	5.7	-3.3	-9.8	-2.4	5.5
MMV1580497	-17.1	5.7	-18.7	-4.1	-8.5	10.0
MMV1580495	-12.2	-0.8	-13.8	3.3	-5.9	7.3
MMV1645152	11.4	0.8	3.3	3.3	4.7	4.0
MMV1633677	-5.7	-3.3	-17.9	-13.0	-10.0	5.8
MMV1593520	5.7	4.1	0.0	8.1	4.5	3.0
MMV1581029	-7.3	4.1	8.9	0.8	1.6	5.9
MMV1580794	-4.9	10.6	1.6	0.0	1.8	5.6
MMV001793	-15.4	-8.9	-7.3	-0.8	-8.1	5.2
MMV251679	-9.8	-5.7	-3.3	0.0	-4.7	3.6
MMV003291	-6.5	4.1	12.2	9.8	4.9	7.2
MMV009948	-12.2	-8.1	3.3	4.9	-3.0	7.3
MMV1174026	-19.5	-14.6	-13.0	-4.1	-12.8	5.6
MMV003820	-12.8	-6.5	-17.1	-17.1	-13.4	4.3
MMV639951	3.3	-3.3	-12.2	2.4	-2.4	6.2
MMV1580491	-10.6	-17.1	-24.1	-22.5	-18.6	5.3
MMV1580492	4.1	0.8	-13.8	-20.3	-7.3	10.1

MMV690621	3.3	-0.8	-20.3	-27.6	-11.4	12.9
MMV1782112	-8.9	-4.9	-15.4	-17.9	-11.8	5.2
MMV1782107	2.4	4.1	-6.5	-13.0	-3.3	6.9
MMV1782106	3.3	4.9	-13.0	-3.3	-2.0	7.0
MMV1782105	-8.1	-6.5	-26.8	-12.2	-13.4	8.0
MMV1782104	-9.8	0.8	-10.6	0.0	-4.9	5.3
MMV1782101	9.8	13.8	4.9	23.6	13.0	6.9
MMV1633969	-0.8	-12.2	-13.8	-6.5	-8.3	5.1
MMV596723	-15.4	-10.6	-3.3	0.0	-7.3	6.1
MMV1581378	-14.6	-1.6	-3.3	4.9	-3.7	7.0
MMV1581031	-9.8	-0.8	0.0	4.1	-1.6	5.0
MMV1580797	-24.4	-19.5	-17.9	-5.7	-16.9	6.9
MMV001761	-16.8	-11.4	-13.0	-0.8	-10.5	5.9
MMV002015	-17.1	-2.4	-5.7	6.5	-4.7	8.4
MMV056052	-13.8	-13.8	-7.3	0.0	-8.7	5.7
MMV1580504	-15.4	-5.7	-8.1	5.7	-5.9	7.6
MMV001961	-11.4	-17.9	-4.1	-7.3	-10.2	5.2
MMV003738	6.5	-4.9	-5.7	-1.6	-1.4	4.8
MMV690540	-22.8	-6.5	-29.3	-36.6	-23.8	11.1
MMV614278	-8.1	-1.6	-28.5	-23.6	-15.4	11.0
MMV1580489	5.7	8.9	-12.2	-20.3	-4.5	12.2
MMV1580499	7.3	8.9	-15.4	-13.0	-3.0	11.2
MMV1782115	*8.1*	1.6	*-8.9*	-11.4	-2.6	7.9
MMV1782114	4.1	-8.1	-17.1	-1.6	-5.7	7.9
MMV018362	2.4	-16.3	-27.6	-4.1	-11.4	11.5
MMV1634556	2.4	22.0	6.5	8.9	10.0	7.3
MMV1634401	9.8	10.6	11.4	11.4	10.8	0.7
MMV1634394	2.4	2.4	-3.3	11.4	3.3	5.2
MMV1633964	-5.7	-2.4	-3.3	3.3	-2.0	3.3
MMV1593516	-9.8	-6.5	-5.7	-20.3	-10.6	5.8
MMV1593513	-5.7	-11.4	0.0	8.1	-2.2	7.2
MMV1593511	-0.3	-0.6	-0.9	7.3	1.4	3.4
MMV637306	-2.8	-3.0	-3.3	0.8	-2.1	1.7
MMV1581377	-3.3	2.4	-4.1	18.7	3.5	9.2
MMV002780	-4.1	-32.5	-4.9	9.8	-7.9	15.3
MMV1580801	-13.0	-11.4	-16.3	5.7	-8.7	8.5
MMV010036	-7.3	-10.6	-7.3	32.5	1.8	17.8
MMV637879	27.6	27.6	31.7	13.0	25.0	7.1
MMV617332	44.8	35.3	7.8	3.4	22.8	17.6
MMV1580480	3.4	6.9	-9.5	-16.4	-3.9	9.5
MMV1580494	-0.9	-3.4	-26.7	-29.3	-15.1	13.0
MMV275100	0.9	-0.9	-20.7	-14.7	-8.8	9.1
MMV1782103	-5.2	-3.4	-23.3	-31.0	-15.7	11.8
MMV1782098	0.0	6.9	-19.8	-21.6	-8.6	12.3
MMV1634397	-2.6	-1.7	-25.0	-21.6	-12.7	10.6
MMV1633963	3.4	23.3	6.9	15.5	12.3	7.7
MMV1593519	8.6	14.7	2.6	15.5	10.3	5.2
MMV002505	9.5	7.8	8.6	6.0	8.0	1.3

MMV1581034	-6.9	-2.6	1.7	4.3	-0.9	4.3
MMV1581033	-6.9	1.7	3.4	12.9	2.8	7.0
MMV1557856	-9.5	-4.3	0.9	25.0	3.0	13.2
MMV1580796	-1.2	-2.1	-4.3	0.7	-1.7	1.8
MMV1580505	-17.2	-19.0	-7.8	4.3	-9.9	9.3
MMV687798	-19.8	-12.1	-8.6	6.0	-8.6	9.4
MMV003297	-13.8	-13.8	7.8	5.2	-3.7	10.2
MMV1580487	1.0	-0.2	-0.9	1.2	0.3	0.8
MMV1580485	-7.8	-2.6	-7.8	6.9	-2.8	6.0
MMV098836	56.0	59.5	21.6	55.2	48.1	15.4
MMV218827	9.5	5.2	-12.1	-15.5	-3.2	10.7
MMV1782111	0.9	-0.9	-25.0	-26.7	-12.9	13.0
MMV1782097	17.2	14.7	0.9	-17.2	3.9	13.7
MMV1634398	12.9	11.2	-3.4	-1.7	4.7	7.4
MMV1634396	-2.6	0.9	-19.0	-26.7	-11.9	11.4
MMV1634385	10.3	-4.3	-15.5	-15.5	-6.2	10.6
MMV1633968	12.9	8.6	-0.9	-3.4	4.3	6.7
MMV688991	6.9	10.3	1.7	2.6	5.4	3.5
MMV1580502	56.0	54.3	49.1	49.3	52.2	3.0
MMV637855	10.3	5.2	0.0	-0.9	3.7	4.5
MMV690467	-0.9	-6.0	-4.3	-2.6	-3.4	1.9
MMV1580493	32.8	39.7	43.1	55.2	42.7	8.1
MMV1580482	2.6	1.7	-1.7	3.4	1.5	2.0
MMV1580501	-31.0	-3.4	-8.6	-2.6	-11.4	11.6
MMV1580496	-22.2	-16.7	-6.9	9.5	-9.1	12.1
MMV1580490	-32.8	-14.7	-5.2	10.3	-10.6	15.6
MMV1782113	-13.8	-14.7	-4.3	12.1	-5.2	10.7
MMV1634393	0.9	-7.8	11.2	-6.0	-0.4	7.5
MMV1634392	-8.6	-4.3	0.0	-0.9	-3.4	3.4
MMV1634389	3.4	15.5	12.1	10.3	10.3	4.4
MMV1581035	20.7	0.0	-12.9	-27.6	-5.0	17.7
MMV1593544	25.0	3.4	-12.9	-25.9	-2.6	19.0
MMV1593521	11.2	4.3	-27.6	-41.4	-13.4	21.8
MMV1593514	16.4	-2.6	-20.7	-13.8	-5.2	14.0
MMV1581036	0.0	2.6	-18.1	-19.8	-8.8	10.2
MMV1581030	1.7	2.6	-16.4	-18.1	-7.5	9.7
MMV247764	-4.3	-4.3	-12.9	-12.9	-8.6	4.3
MMV019724	69.8	74.1	66.4	68.1	69.6	2.9
MMV638198	44.0	48.3	34.5	47.4	43.5	5.5
MMV637413	-0.9	-7.8	-10.3	-8.6	-6.9	3.6
MMV658803	-28.4	-22.4	-24.1	-22.4	-24.4	2.5
MMV1580500	-9.5	-1.7	-4.3	3.4	-3.0	4.7
MMV1580498	-19.0	-29.3	-34.5	-24.1	-26.7	5.8
MMV1580488	30.2	38.8	35.3	45.7	37.5	5.6
MMV1580486	-4.5	-2.3	-6.4	-4.3	-4.4	1.4
MMV1782222	-0.9	-4.3	6.0	12.9	3.4	6.6
MMV690706	-22.4	-12.9	-8.6	0.0	-11.0	8.1
MMV1782220	-1.7	-3.4	11.2	18.1	6.0	9.0

MMV1782213	1.7	-13.8	-5.2	0.0	-4.3	6.0
MMV1782214	15.5	25.9	12.1	4.3	14.4	7.7
MMV1782211	49.1	39.7	25.0	17.2	32.8	12.4
MMV1782210	-20.7	-3.4	-22.4	-19.0	-16.4	7.6
MMV1782208	-5.2	-9.7	-9.0	-6.4	-7.5	1.8
MMV1006203	-12.9	-2.6	-7.8	-12.1	-8.8	4.1
MMV1782353	-15.5	-6.9	-15.5	-6.9	-11.2	4.3
MMV1782349	-6.0	-0.9	-9.5	4.3	-3.0	5.2
MMV1782355	-19.0	-6.9	-17.2	-7.8	-12.7	5.4
MMV1782352	-16.4	-5.2	-24.1	-8.6	-13.6	7.3
MMV1782350	25.9	24.1	26.7	29.3	26.5	1.9
MMV1782386	2.6	0.9	-14.7	-6.9	-4.5	6.9
MMV1782412	6.9	-2.6	-3.4	-6.9	-1.5	5.1
MMV1613563	0.0	-0.9	-6.9	-19.8	-6.9	7.9
MMV1580843	-17.2	-12.1	-25.9	-16.4	-17.9	5.0
MMV690555	-30.2	-36.2	*-12.9*	*-23.3*	-25.6	8.7
MMV1613559	-36.2	-10.3	-8.6	-11.2	-16.6	11.4
MMV1634361	-12.1	-3.4	-11.2	-8.6	-8.8	3.4
MMV002350	-11.2	-2.6	2.6	-6.0	-4.3	5.0
MMV1580483	2.6	-5.2	-3.4	-15.5	-5.4	6.5
MMV1782209	0.0	-4.3	-12.9	-6.0	-5.8	4.7
MMV003069	-7.8	-3.4	-14.7	-22.4	-12.1	7.2
